# Environmentally Oriented Analysis of Benefits and Expenditures in the Life Cycle of a Wind Power Plant

**DOI:** 10.3390/ma16020538

**Published:** 2023-01-05

**Authors:** Łukasz Sobaszek, Izabela Piasecka, Józef Flizikowski, Andrzej Tomporowski, Edgar Sokolovskij, Patrycja Bałdowska-Witos

**Affiliations:** 1 Faculty of Mechanical Engineering, Lublin University of Technology, 20-618 Lublin, Poland; 2 Department of Machines and Technical Systems, Faculty of Mechanical Engineering, University of Sciences and Technology in Bydgoszcz, Al. Prof. S. Kaliskiego 7, 85-796 Bydgoszcz, Poland; 3 Department of Automobile Engineering, Faculty of Transport Engineering, Vilnius Gediminas Technical University, J. Basanaviciaus str. 28B, LT-03224 Vilnius, Lithuania

**Keywords:** wind farms, renewable energy sources, Impact 2002+, life cycle assessment

## Abstract

The motivation for this study was the need to extend and supplement the previously conducted research on technical objects in the renewable energy sector with analyses of the environmental impact of the production, operation and post-operational development stages of the wind power plant. The main purpose of the work was to investigate, analyze and assess the ecological effects of a real facility, which is a 2 MW Vestas V90/105 m wind farm, throughout its life cycle. The life cycle assessment analysis of the 2 MW wind power plant was performed using Impact 2002+ modeling. The results are presented for all impact levels and categories. The production stage was characterized by the highest total level of harmful effect. The use of recycling reduces the negative impacts of the life cycle by 6.5%. The investigated technical facility has the greatest negative impact during the production stage, especially in the area of depletion of fossil resources and human health.

## 1. Introduction

Global energy demand depends on many factors—the most important of them include the pace of the economic development of individual countries, population growth, the evolution of social structures, technical progress in the use of devices and the creation of new technical solutions [1,2,3,4,5]. The modern economy is based primarily on the consumption of goods and services. Commodities necessary for the functioning of society include the electricity, heat, and energy contained in liquid and solid fuels. A significant part of the total energy demand in the world is for electricity, without which it is difficult to imagine the existence of any area of life [6,7,8,9]. 

The ever-growing demand for electricity forces an increase in its production. The main sources of energy in the world are fossil fuels: coal and lignite, crude oil and natural gas. Unfortunately, the extraction of conventional fuels and the extraction of the energy contained in them is associated with the deterioration of the quality of the natural environment caused by the emission of harmful substances. The biggest problem is the emission of carbon dioxide accompanying the burning of fossil fuels, which, according to many scientists, largely contributes to the deepening of the greenhouse effect and the warming of the climate. According to one source [10], approximately 8 billion tons of CO_2_ reach the atmosphere every year, and the amount will increase. Other negative impacts of traditional energy generation include the resulting waste, such as dust, ash, sulfur and nitrogen oxides, as well as heavy metals. Each of the emitted compounds cause the deterioration of the condition of the environment to a greater or lesser extent, and the tangible effects of their adverse impact include the contamination of water and soil, acid rains and climate changes [11,12,13,14,15,16,17]. 

Due to the environmental harmfulness and the forecasts of the depletion of natural resources of fossil fuels (the most optimistic of them assume coal extraction for about 200 years, oil for about 100 years and gas for 150 years [18]), alternative energy resources are sought. A good solution seems to be obtaining energy from available, naturally occurring sources of renewable energy, such as the wind, the sun, biomass, water, earth heat or sea tides [19,20,21,22].

The use of renewable sources for energy production will undoubtedly contribute to the reduction of the emission of toxic compounds negatively affecting the ecosystem. The most important other positive aspects of obtaining energy from alternative sources are their renewable, unlimited resources, the flexibility of location and their ability to work in separate systems or, in limited circumstances, be connected to the power grid.

Increasing the share of renewable energy sources (RES) in European or Polish conditions requires certain financial outlays and changes in the structure of the power system that would enable the transition from central energy production and its distribution over long distances towards distributed energy sources located near recipients. Wind farms fit into the implementation of this strategy [23,24,25,26,27].

Generating energy in wind farms is considered ecologically clean due to the emission-free conversion of wind energy into electricity. The analyses conducted thus far of the impact of wind farms on the environment mainly concern their visible impact at the stage of use, which includes, inter alia, their influence on migrating birds, emission of vibrations, audible noise and infrasound, as well as their influence on the surrounding landscape. On the other hand, less attention is paid to the benefits, inputs and impacts of the life cycle of a wind power plant. Significant amounts of various materials, e.g., steel, plastics, concrete, are used for the production of wind turbine components, and large amounts of energy are also used. At the time of the liquidation of the working unit of a wind power plant, the materials used for its production should be disposed of after use, e.g., by landfill or recycling. The production and post-use management stages of the life cycle of a wind power plant are therefore not insignificant in the assessment of its total environmental impact. It is worth considering whether the benefits of wind farm operation in the use phase, in the form of the electricity generated and the reduction of greenhouse gas emissions, exceed the environmental and energy inputs in the remaining stages of its life cycle [28,29,30,31]. 

Following the global trends, it was decided to investigate the intensively developing area of wind energy. Thanks to the analysis, assessment and study of the environmental potential in the life cycle of one selected type of wind farm, it will be possible to effectively assess the cycle of the production, use and management of its potentials.

The rapid development of wind energy and other renewable energy sources requires, apart from the energy and economic aspects, an analysis of their impact on the environment. The concept of sustainable economic development requires taking into account the environmental aspects of the existence of the required technical facilities.

The research part of the work presents the basic assumptions and indicators and the program. The detailed characteristics of the tested and assessed plastics, materials and elements, as well as information on the produced energy and environmental energy consumption, are also approximated. 

There are few studies in the world literature in which life cycle analyses of wind power plants were carried out using the Impact 2002+ method. Most of the research conducted focuses solely on the impact of the plants’ life cycle on the GWP (Global Warming Potential), ignoring other negative impacts of the systems considered, which reduce the quality of the environment, pose a threat to human health and increase the depletion of raw materials, factors that also require detailed analysis, especially in the context of the sustainable development of energy systems. Unfortunately, analyses using the LCA methodology are still not very popular in Poland. In this study, an attempt to outline the local perspective on the issue of the impact of selected renewable energy sources on the environment was made. Hence, the decision was made to carry out a real case study.

The main aim of the work is to develop and implement a methodology for researching the ecological expenditure of a wind power plant throughout its life cycle, starting from its production, through its use, to the management of its potentials, including mainly raw materials, plastics and other materials. 

## 2. Materials and Methods

The durability of wind farms is estimated at about 25 years [31]. The following three stages were adopted for the life cycle of the tested wind farm: design—shaping the resulting idea, use—the main stage of achieving the assumed goals of existence, and post-utility management—ending the life cycle. In the manufacturing process of a wind power plant, the initial idea takes on real material dimensions with the assumed properties. Important for this phase, in the context of environmental impact, is the appropriate organization and selection of optimal production processes characterized by low emissions of pollutants and waste. Usage processes are the main and the most important stage in the life cycle of a wind power plant, as it is then then that the goals for which the plant was built are realized. Power plant utilization covers all processes related to the use of a technical object in accordance with its intended purpose [32,33,34,35,36]. The last stage ending the life cycle of a wind power plant is post-utility management, which for a time was not taken into account as a separate phase in the existence of technical systems. The problems with the generated waste and the related environmental threats contributed to a change in the perception of post-use management issues, which are now considered among those important from the point of view of the rational use of materials, minimizing energy consumption and reducing environmental degradation. This resulted in the recycling of wind turbine components and the re-use of suitable components [34,35,37].

A life cycle assessment was carried out for an onshore, three-blade horizontal axis wind power plant with a capacity of 2 MW located in central Poland. Assessment of the life cycle of the plastics, materials and other components of renewable energy systems is possible thanks to the use of various models, including Life Cycle Assessment. Environmental LCA was chosen as the method of assessing the potential impact of the wind power plant on human health, ecosystem quality and resource depletion. In accordance with ISO 14040 (Environmental management, Life cycle assessment, Principles and framework) and ISO 14044 (Environmental management, Life cycle assessment, Requirements and guidelines) standards, the LCA analysis performed in this work included four stages: determination of the goal and scope, life cycle inventory (LCI), life cycle impact assessment (LCIA) and interpretation [38,39].

The research began with the determination of the goal and scope. Based on the previous analysis of the current state of knowledge and technology, it was found that the literature lacks, in context, a detailed assessment of the life cycle of wind power plants [38,40,41]. The data used in the analysis were obtained from the producers of the renewable energy systems under consideration or were downloaded from the databases of the SimaPro software. The time range of the analysis is twenty-five years. The geographical scope of this research is the area of Europe, because the company that provided the data operates on the entire European market. The cut-off level established for the research was 0.1%. The analysis conducted should be classified as bottom-up and was mainly used to describe the existing situation (retrospective analysis), but also to create more pro-environmental solutions (prospective analysis). The level of advancement of the analysis classifies it among detailed analyses. The functional unit is the productivity of the researched object, which is 5325 MWh/year [38,42].

Based on the previous analysis of the state of knowledge and technology, it was acknowledged that the literature needs a detailed life cycle assessment of a wind power plants with particular attention to budget and to the benefits of their life cycle. It was also considerably important when articulating the goal and scope to accumulate as much and the best possible quality data on the objects of analysis as possible. This was possible thanks to cooperation with a company that produces materials and elements for wind power plants, which has a leading position on the European and domestic market. Due to the conclusion of a data confidentiality agreement with the company producing the RES system analyzed, this study does not disclose all detailed information on the structure of the analyzed subjects and their technological data [38,43,44].

The total weight of the elements and materials of the tested wind power plant is about two thousand tons. The foundations account for the largest share of the mass of the building—roughly 79% (of which roughly 96% is concrete and 4% is steel). Among the other most important elements of the power plant, one can distinguish the tower, contributing roughly 15% of the weight of the entire object (mostly made of steel), the nacelle, with a roughly 4% share (its elements are mostly made of cast iron—roughly 49% of the nacelle weight, steel—roughly 38%, aluminum—roughly 4%, polymer materials—roughly 3% and copper—roughly 2%) and the rotor, with a roughly 2% share (around 50% of this weight is a hub made mainly of nodular cast iron, and the other 50% comes blades made of polymers reinforced with fiberglass) (Table 1) (data obtained from the manufacturers, the investor and the producers).

In the next step, a detailed analysis of the life cycle of the technical objects under investigation was created. The necessary simulation examination was conducted using SimaPro software and IMPACT 2002+ calculation processing. The acquired results and their rendition are presented in Section 3. The last part of the analysis, including the rendition of the acquired results, is presented in Section 3, Section 4 and Section 5.

The Impact 2002+ model is a combination of two methods—CML and Eco-indicator 99—and groups similar categories of intermediate points to endpoints. It is primarily used to calculate the maximum negative environmental impact that may occur in the territory of Europe. The spheres of evaluation, the technosphere and the ecosphere influence the results obtained by this model. Importantly, the model functions in the technical space, and the obtained results indicate damage caused in the ecological space.

The model of a wind power plant system as a research object is a representation of the actual and probable states of its environmental functioning. It is created as the result of analyses and studies or is imposed by the modeler.

The object of experimental research in this work was a Vestas V90/105 m wind power plant, operating and connected with its surroundings (environment). 

## 3. Results

The results of the implementation of this stage for one life cycle of the Vestas V90/105 m wind farm, sorted by their impact categories, are presented in Table 2, Table 3, Table 4, Table 5, Table 6, Table 7, Table 8, Table 9 and Table 10. The results of the impacts were given in three units characteristic for Impact 2002+ modeling in the LCA method: PDF m^2^/r, DALY and MJ. The cut-off level for all analyses was 0.05%.

The results characterizing the environmental consequences occurring in the life cycle of the Vestas V90/105 m wind farm showed a particularly high level of negative impact during the production stage in the category of inorganic compounds causing respiratory diseases (7.33 DALY), processes related to the extraction of minerals (1,280,000 MJ) and non-renewable energy (2,470,000 MJ). Recycling would make it possible to minimize the harmful impact of the life cycle of the analyzed wind farm in most impact categories, including inorganic compounds causing respiratory diseases (−0.143 DALY), processes related to the extraction of minerals (−31,300 MJ) and non-renewable energy (−374,000 MJ) (Table 2).

Table 3 presents the results characterizing the environmental consequences for carcinogenic compounds occurring in the life cycle of the Vestas V90 wind farm. The conducted research revealed a particularly high level of negative interactions at the production stage for arsenic (0.162 DALY) and cadmium (0.042 DALY) ions. The influence of cadmium (0.011 DALY), arsenic (0.006 DALY) and polycyclic aromatic hydrocarbons (0.003 DALY) ions was also significant. Recycling processes would reduce the harmful effects for arsenic (−0.073 DALY) and cadmium (−0.005 DALY) ions. A particularly high level of destructive impact during post-use management in the form of landfill (1.545 DALY) is visible. The use of recycling processes would reduce the harmful impact of the life cycle of the investigated wind farm by −0.079 DALY. 

Table 4 summarizes the results characterizing the environmental consequences for organic compounds that cause respiratory diseases occurring at individual stages of the Vestas V90 wind farm life cycle. The analyses performed indicate a significantly high level of negative impact for non-methane volatile organic compounds during the production of the tested technical object (0.003 DALY). Methane (7.90 × 10^−5^ DALY), ethene (3.74 × 10^−5^ DALY), pentane (2.33 × 10^−5^ DALY) and butane (1.64 × 10^−5^ DALY) also play an important role in shaping the impacts of this category, as to xylene (1.45 × 10^−5^ DALY), fossil methane (1.12 × 10^−5^ DALY) and propane (1.03 × 10^−5^ DALY). In this case, recycling makes it possible to significantly reduce the harmful effects of non-methane volatile organic compounds (−6.42 × 10^−4^ DALY) and methane (−3.91 × 10^−5^ DALY). A particularly high level of negative impact during the production phase is evident (0.003 DALY). The use of recycling processes will make it possible to reduce the harmful impact of the life cycle of the tested wind farm by −0.001 DALY.

The results of the studies characterizing the environmental consequences for inorganic compounds causing respiratory diseases in the life cycle of the Vestas V90 wind farm are summarized in Table 5. The highest levels of harmful effects in this category for the production stage were produced by sulfur oxide (6.405 DALY), medium level—solid particles < 10 µm (0.439 DALY), nitric oxide (0.39 DALY), sulfur dioxide (0.03 DALY) and solid particles < 2.5 µm (0.026 DALY). The use of recycling will make it possible to reduce the negative impact of the life cycle of the wind processor, especially by reducing emissions of sulfur oxide (−0.066 DALY) and nitrogen oxide (−0.065 DALY). The production stage had the highest level of negative environmental impact (7.33 DALY).

The results characterizing the environmental consequences of the ionizing radiation occurring in the various stages of the life cycle of the Vestas V90 wind farm are presented in Table 6. The highest level of harmful effects during the production stage was caused by the radon 222Rn isotope (0.0162 DALY), the carbon isotope 14C (0.0018 DALY) and the isotope cesium 137Cs (0.001 DALY). There is a visibly high level of negative impact for the production stage: 0.0193 DALY.

Table 7 presents the results characterizing the environmental consequences for ozone layer depletion occurring in the various stages of the life cycle of the Vestas V90 wind farm. The analyses conducted revealed a significantly high detrimental effect in the production stage, especially for bromotrifluoromethane (0.001 DALY) and 1,2-dichloro-1,1,2,2-tetrafluoroethane (0.0001 DALY) in this category of the environmental impacts of the investigated technical object. The highest level of harmful impact occurs during the production stage due to the production of the plastics, materials and wind turbine components (0.0012 DALY). Recycling will make it possible to minimize negative impacts throughout the life cycle in the analyzed category by a total of −0.0001 DALY.

Table 8 presents the results characterizing the environmental consequences for the processes related to land occupation, occurring in the various stages of the life cycle of the Vestas V90 wind farm. The very high level of negative impacts in this category for the production stage was particularly visible, mainly in the form of the use of class II-III land (2.58 × 10^4^ PDF m^2^/y), the conversion of land into the area of mineral resource extraction (8.35 × 10^3^ PDF m^2^/y), the use of class II-IV land (3.25 × 10^3^ PDF m^2^/y), the use of III-IV class land (3.19 × 10^3^ PDF m^2^/y) and its transformation into artificial water reservoirs (1.97 × 10^3^ PDF m^2^/y). The highest level of harmful impact was recorded for the production process (3.34 × 10^4^ PDF m^2^/y), while the levels are significantly lower for the use stage (3.96 × 10^3^ PDF m^2^/y) and landfilling (2.92 × 10^3^ PDF m^2^/y).

The results characterizing the environmental consequences for the processes related to the extraction of minerals occurring in each stage of the life cycle of the Vestas V90 wind farm are presented in Table 9. The analyses conducted showed a particularly high, detrimental effect on the surroundings for the production stage of the technical object, mainly caused by processes related to the extraction of fossil nickel (1.04 × 10^6^ MJ), fossil copper (8.31 × 10^4^ MJ), and co-combustible bauxites (8.08 × 10^4^ MJ)) and fossil chromium (4.95 × 10^4^ MJ). The production stage of the plastics, materials and elements of a wind turbine is characterized by the highest negative impact on the environment (1.28 × 10^6^ MJ). The choice of recycling as a method of post-consumer management will reduce the total harmful impact of the life cycle by −3.13 × 10^4^ MJ. 

Table 10 presents the results of the studies characterizing the environmental consequences for the processes related to the extraction of non-renewable energy, occurring in the various stages of the life cycle of the Vestas V90 wind farm. The highest level of harmful impacts is characteristic of the processes related to the production of wind turbine components, including the extraction of crude oil, requiring 42.6 MJ/kg of non-renewable energy (1.3 × 10^6^ MJ) and natural gas, requiring 35 MJ/m^3^ of non-renewable energy (6.11 × 10^5^ MJ). A significantly high level of destructive impact on the environment was recorded for the production stage of the wind turbine (2.47 × 10^6^ MJ). Reducing the negative impact of the existence cycle is possible through the use of recycling processes (−3.74 × 10^5^ MJ). 

**Figure 1:**
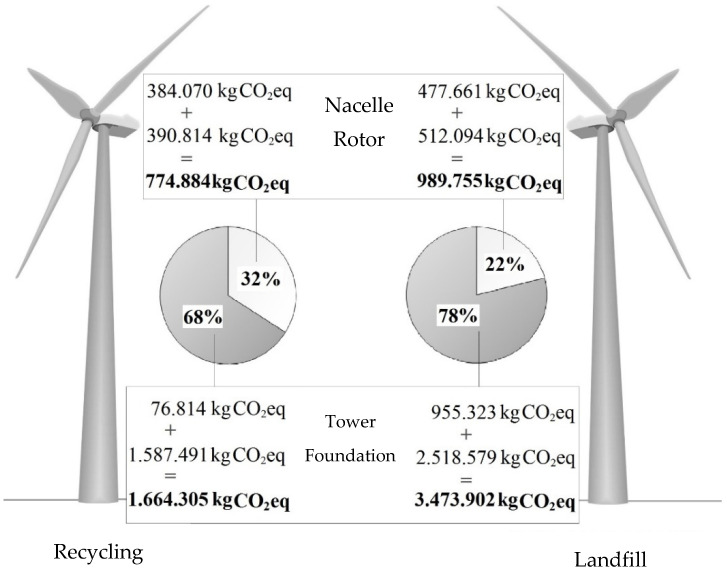
Greenhouse gas emissions in the life cycle of selected elements of a wind power plant [own research].

Figure 1 shows the amount of greenhouse gas emissions in the life cycle of selected elements of the Vestas V90/105 m wind farm. In this case, two options for post-use management were adopted, landfill or recycling, and the structure of the power plant was divided into the same four main parts: nacelle, rotor, tower and foundation. The foundations (2,518,579 kg CO_2eq_) were the highest in the lifecycle of greenhouse gas management in the form of landfill storage. The share of the mobile above-ground part of the power plant (nacelle + rotor) was smaller than that of the fixed part (tower + foundations) in the total negative impact on the surroundings of the entire analyzed power plant and amounted to 22%. On the other hand, assuming that the post-use development of the research object will take the form of recycling, it can be noticed that the greatest amount of GHG is also found in the foundations (1,587,491 kg CO_2eq_). In this case, the share of the mobile part of the above-ground wind turbine is about 1/3 of the total harmful effect of the Vestas V90/105 m life cycle. 

## 4. Discussion

The aim of the research in this study was to assess the impact of the life cycle of the Vestas V90 wind farm on the environment. The research was performed using the LCA method and the Impact 2002+ model by means of the SimaPro 7.1 software (Pré Consultants B.V., Amersfoort, The Netherlands). To analyze the impact of the entire wind farm, various categories of impacts and the values of emissions of harmful compounds into the soil, water and atmosphere were taken into account. The cut-off level for all analyses was 0.05%. 

LCA analysis in the field of wind energy initially focused on power plants with a capacity of less than one MW. Schleisner [45] concluded one of the first examinations of a 500 kW turbine, whilst Ardente et al. [46,47] carried out a study on a wind power plant consisting of 11 turbines with a capacity of 660 kW each. There are also studies devoted to local issues: Martínez et al. [48] studied the impact of the wind power plant life cycle on the environment in Spain, Schleisner [45]—in Denmark, Wagner et al. [49]—in Germany, Ardente et al. [46]—in Italy, Al-Behadili and El-Osta [50]—in Libia, Oebelsa et al. [51]—in Brasil, Kabir et al. [52]—in Canada, Alsaleh et al. [53]—in United States and Vargas et al. [54]—in Mexico. In addition, plenty of studies were also performed on wind energy systems with high installed capacities. For example: Alexandra et al. performed LCA tests for two offshore and two onshore wind power plants [47]. In the case of this analysis, the local conditions in Poland were considered. However, there are very few studies in the world literature in which the analysis of the life cycle of wind power plants was executed using the IMPACT 2002+ method. Most of the existing examinations are focused only on the impact of the life cycle of the power plant on GWP (Global Warming Potential), disregarding other negative impacts on the quality of the environment and human health and the depletion of natural resources, which also require detailed analysis, especially from the perspective of the sustainable development of energy systems. Oebels et al. [51] investigated a 1.5 MW power plant and found that the life cycle of its steel tower was the primary reason for its highest greenhouse gas emissions. Kabir et al. [52] examined three models of wind turbines of different power, realizing that the more power a turbine has, the lesser its CO_2_ emissions per kWh of generated energy. Chipindula et al. [55] carried out an LCA of offshore and onshore wind power plants with various capacities installed, resulting in the confirmation that an increase of the mentioned capacity translates into a decrease in carbon dioxide emissions per amount of electricity produced. Furthermore, Alsaleh et al. [53] examined a 2 MW turbine, taking into account various periods of the operation of this type of object, resulting in the conclusion that the production stage causes the highest GHG emissions into the atmosphere.

The highest level of harmful impact during the production of the Vestas V90/105 m wind farm was recorded in the categories of inorganic compounds causing respiratory diseases (7.33 DALY), processes related to non-renewable energy (2.47 × 10^6^ MJ) and the mining of minerals (1.28 × 10^6^ MJ). For the use phase, these were the key categories: non-renewable energy-related processes (2.09 × 10^5^ MJ), inorganic compounds causing respiratory diseases (3.38 × 10^−1^ DALY) and carcinogens (1.06 × 10^−1^ DALY). However, in the case of post-use management in the form of landfill storage, the significant categories included carcinogenic compounds (1.55 DALY). The choice of recycling as a form of management makes it possible to reduce the negative impact of the life cycle of the plant, especially in the area of processes related to the acquisition of non-renewable energy (−3.74 × 10^−5^ MJ) and inorganic compounds causing diseases respiratory system (−1.43 × 10^−1^ DALY). The production stage was characterized by the highest total level of harmful effects. The use of recycling reduces the negative impacts of the life cycle by 6.5%. The technical facility has the greatest negative impact during the production stage, especially in the areas of the depletion of fossil resources and effects on human health. 

The highest level of harmful emissions occurring in each stage of the life cycle of the Vestas V90/105 m wind farm was recorded with regard to the atmospheric environment, which was most influenced by the production stage; a medium level was recorded with regard to the aquatic environment, and the lowest with regard to the soil environment (the most significant influence of the production stage). 

The term eco-design is defined as “the integration of environmental aspects into product design and development with the aim of reducing adverse environmental impacts throughout the whole product’s life cycle” (ISO, 2011, ISO/TR 14062, 2012) [56]. Materials, elements, work teams and entire technical objects created in this way should be modeled on the life cycle naturally occurring in nature, which is an ideal example of a closed circulation of matter. The environmental aspects that should be analyzed in order to be able to talk about the introduction of eco-designing principles in the life cycle of wind power plants include, for example, issues such as:

− use of materials with the lowest possible negative impact on the environment for production,
− using fewer resources during the production process,
− reduction of the amount of pollution and by-products,
− ensuring an adequate service life,
− facilitating the re-use of materials, components and/or workgroups,
− striving to reduce the impact of post-consumer development on the environment.

As previously indicated, one of the main techniques used in eco-design is Life Cycle Assessment. The LCA studies carried out as part of this study enable the identification of both individual chemical compounds and substances, as well as materials, components, working units or entire stages of the life cycle of wind power plants, characterized by the greatest negative impact on the environment. This identification will make possible the introduction of changes in the subsequent cycles of newly designed wind power plants, in line with the key assumptions of eco-design and sustainable development.

## 5. Conclusions

In the light of the analysis of our research results and conclusions, the research goal of the work, which consisted of determining the potentials of environmental influences in the life cycle of the tested working machine, its productivity, expenditures and the environmental impact of the research object, has been achieved.

The main goal of the work was achieved by means of the development and implementation of a methodology for the research and evaluation of ecological expenditure in the life cycle of a wind power plant, starting from its generation, through its use, to the development of its potentials. 

The LCA method was used to determine the environmental inputs of the research object, and within its framework the Impact 2002+ procedure was used as a tool for determining the environmental inputs of greenhouse gas emissions. 

Among the possible actions to reduce selected inputs, our study suggests:

− development and implementation by manufacturers of wind turbines using technology that allows for the replacement of faster-wearing elements, e.g., nacelles and rotors or their parts after the first life cycle, while leaving slower-wearing elements, e.g., foundations and towers, for the second life cycle, as this will allow for the second life cycle to reduce environmental inputs;
− use of pro-environmental construction materials;
− designing structures that enable easy post-use separation of materials and elements;
− minimization of energy consumption, material consumption and emission intensity of production processes, use and post-consumer disposal of materials, materials and elements of a wind power plant;
− optimization at the stage of designing the consumption of the materials (mainly steel) used to build wind farms that have the highest environmental impact;
− development of comprehensive, pro-environmental standards regarding the method of post-consumer management of plastics, materials and elements of a wind power plant.

Due to significant differences in its impact on the environment depending on the form used (road, rail, sea or air transport) and the distances that must be covered for different locations both in relation to Poland, Europe and the world, the wind turbine transport stage was not included in the study.

In the future, it is planned to extend the conducted research to include other types of wind power plants (including offshore wind power plants) and to conduct LCA analyses of other renewable energy sources.

## Figures and Tables

**Table 1:** Register of materials for the production of the Vestas V90/105 m wind power plant [own study].

No.	Element	Material	Weight [kg]	% Content in Wind Power Plant	Aluminum [kg]	Copper [kg]	Steel [kg]	Cast Iron [kg]	Polymer Materials [kg]	Concrete [kg]	Oil [kg]	Other [kg]	Data Source
1	NACELLE												
1.1	Generator with a cooler	copper	1430	0.08	x	1430	x	x	x	x	x	x	Vestas
		cast iron	3920	0.22	x	x	x	3920	x	x	x	x	
		steel	2090	0.12	x	x	2090	x	x	x	x	x	
		other	60	0.00	x	x	x	x	x	x	x	60	x
		total: generator	7500	0.43	x	x	x	x	x	x	x	x	Vestas
1.2	Gearbox	high-quality steel	2620	0.15	x	x	2620	x	x	x	x	x	Vestas
		cast iron	14060	0.80	x	x	x	14,060	x	x	x	x	
		oil	280	0.02	x	x	x	x	x	x	280	x	
		other	40	0.00	x	x	x	x	x	x	x	40	x
		total: gearbox	17,000	0.97	x	x	x	x	x	x	x	x	Vestas
1.3	Transformer	steel	4150	0.24	x	x	4150	x	x	x	x	x	Siemens
		aluminum	860	0.05	860	x	x	x	x	x	x	x	
		other	80	0.00	x	x	x	x	x	x	x	80	x
		total: transformer	5090	0.29	x	x	x	x	x	x	x	x	Siemens
1.4	Main shaft with body	high-quality steel	9520	0.54	x	x	9520	x	x	x	x	x	own measurement
		cast iron	1795	0.10	x	x	x	1795	x	x	x	x	
		other	20	0.00	x	x	x	x	x	x	x	20	x
		total: main shaft	11,335	0.65	x	x	x	x	x	x	x	x	own measurement
1.5	Coolers	aluminum	960	0.05	960	x	x	x	x	x	x	x	own measurement
1.6	Hydraulic system	oil	360	0.02	x	x	x	x	x	x	360	x	own measurement
		steel	2660	0.15	x	x	2660	x	x	x	x	x	
		aluminum	420	0.02	420	x	x	x	x	x	x	x	
		other	200	0.01	x	x	x	x	x	x	x	200	x
		total: hydraulic system	3640	0.21	x	x	x	x	x	x	x	x	own measurement
1.7	Switchboards, converters, connections	steel	340	0.02	x	x	340	x	x	x	x	x	own measurement
		copper	240	0.01	x	240	x	x	x	x	x	x	
		aluminum	180	0.01	180	x	x	x	x	x	x	x	
		other	120	0.01	x	x	x	x	x	x	x	120	x
		total: switchboards and converters,	880	0.05	x	x	x	x	x	x	x	x	own measurement
1.8	Nacelle platform	cast iron	14,590	0.83	x	x	x	14,590	x	x	x	x	Vestas
		steel	3750	0.21	x	x	3750	x	x	x	x	x	
		total: nacelle platform	18,340	1.04	x	x	x	x	x	x	x	x	
1.9	Nacelle case	steel	1100	0.06	x	x	1100	x	x	x	x	x	own measurement
		polymer materials	1815	0.10	x	x	x	x	1815	x	x	x	
		other	340	0.02	x	x	x	x	x	x	x	340	x
		total: nacelle case	3255	0.19	x	x	x	x	x	x	x	x	own measurement
Total: nacelle			68,000	3.87	2420	1670	26,230	34,365	1815	0	640	860	Vestas
2	ROTOR												
2.1	Blades	steel	1750	0.10	x	x	1750	x	x	x	x	x	Vestas
		polymer materials	18,250	1.04	x	x	x	x	18,250	x	x	x	
		total: blades	20,000	1.14	x	x	x	x	x	x	x	x	
2.2	Hub	cast iron	17650	1.00	x	x	x	17,650	x	x	x	x	Vestas
		polymer materials	200	0.01	x	x	x	x	200	x	x	x	
		other	150	0.01	x	x	x	x	x	x	x	150	x
		total: hub	18,000	1.02	x	x	x	x	x	x	x	x	Vestas
Total: rotor			38,000	2.16	0	0	1750	17,650	18,450	0	0	150	Vestas
3	TOWER												
3.1	Tower rings	steel	253,775	14.45	x	x	253775	x	x	x	x	x	Vestas
		aluminum	1295	0.07	1295	x	x	x	x	x	x	x	
		copper	380	0.02	x	380	x	x	x	x	x	x	
		other	550	0.03	x	x	x	x	x	x	x	550	x
Total: tower			256,000	14.57	1295	380	253,775	0	0	0	0	550	Vestas
4	FOUNDATIONS												
4.1	Reinforcement	steel	54,545	3.10	x	x	54,545	x	x	x	x	x	construction project
4.2	Concrete	cement	209,050	11.90	x	x	x	x	x	x	x	x	
		aggregate	1,036,775	59.01	x	x	x	x	x	x	x	x	
		water	90,400	5.15	x	x	x	x	x	x	x	x	
		other	3390	0.19	x	x	x	x	x	x	x	x	
		total: concrete	1,339,615	76.25	x	x	x	x	x	133,961	x	x	
4.3	Other		650	0.04	x	x	x	x	x	x	x	650	x
Total: foundations			1,394,810	79.39	0	0	54,545	0	0	1,339,615	0	650	construction project
TOTAL: WIND POWER PLANT			1,756,810	100.00	3715	2050	336,300	52,015	20,265	1,339,615	640	2210	x
			% content of materials in wind power plant		0.21	0.12	19.14	2.96	1.15	76.25	0.04	0.13	

**Table 2:** The results characterizing the environmental consequences occurring at individual stages of the Vestas V90 wind farm life cycle, taking into account the impact categories [own research].

Impact Category	Unit	Production	Use	Landfill	Recycling
Carcinogens	DALY	2.25 × 10^−1^	1.06 × 10^−1^	1.55	−7.94 × 10^−2^
Respiratory organics	DALY	2.86 × 10^−3^	2.23 × 10^−4^	5.07 × 10^−4^	−6.77 × 10^−4^
Respiratory inorganics	DALY	7.33	3.38 × 10^−1^	3.55 × 10^−2^	−1.43 × 10^−1^
Ionizing radiation	DALY	1.93 × 10^−2^	3.19 × 10^−4^	2.97 × 10^−4^	0.00
Ozone layer depletion	DALY	1.17 × 10^−3^	1.59 × 10^−5^	5.91 × 10^−6^	−8.72 × 10^−5^
Land use	PDF·m^2^/r	3.34 × 10^4^	3.96 × 10^3^	2.92 × 10^3^	0.00
Minerals	MJ	1.28 × 10^6^	2.91 × 10^4^	1.04 × 10^3^	−3.13 × 10^4^
Non-renewable energy	MJ	2.47 × 10^6^	2.09 × 10^5^	6.65 × 10^4^	−3.74 × 10^5^

**Table 3:** The results of the studies characterizing the environmental consequences for carcinogenic compounds occurring in the various stages of the life cycle of the Vestas V90 wind farm, DALY [own research].

Substance	Influence Area	Production	Use	Landfill	Recycling
Arsenic	Air	5.68 × 10^−3^	1.12 × 10^−3^	8.59 × 10^−5^	×
Benzo (α) pyrene	Air	1.68 × 10^−4^			×
Cadmium	Air	4.16 × 10^−2^	1.97 × 10^−3^	3.59 × 10^−4^	−4.77 × 10^−3^
Dioxins, measured as 2,3,7,8-tetrachlorodibenzo-p-dioxin (TCDD)	Air	2.74 × 10^−4^	×	×	×
Metals, unspecified	Air	1.28 × 10^−4^	×	×	3.97 × 10^−3^
Nickel	Air	7.32 × 10^−4^	×	×	−2.22 × 10^−5^
PAHs, polycyclic aromatic hydrocarbons	Air	3.98 × 10^−4^	×	6.56 × 10^−6^	−2.52 × 10^−5^
Solid particles, <2.5 µm	Air	3.62 × 10^−4^	9.35 × 10^−2^	×	×
Arsenic, ions	Water	1.62 × 10^−1^	7.93 × 10^−3^	7.03 × 10^−2^	−7.33 × 10^−2^
Cadmium, ions	Water	1.12 × 10^−2^	2.13 × 10^−4^	1.47	−2.09 × 10^−3^
Metal ions, unspecified	Water	2.24 × 10^−5^	×	×	−2.95 × 10^−3^
PAHs, polycyclic aromatic hydrocarbons	Water	2.66 × 10^−3^	×	×	−2.15 × 10^−4^
Arsenic	Soil	7.92 × 10^−5^	×	×	×
Cadmium	Soil	×	2.13 × 10^−4^	×	×
	DALY	0.225	0.106	1.545	−0.794

**Table 4:** The results of the studies characterizing the environmental consequences for organic compounds causing respiratory diseases for individual stages of the life cycle of the Vestas V90, DALY [own research].

Substance	Influence Area	Production	Use	Landfill	Recycling
Acetaldehyde	Air	1.30 × 10^−6^	×	×	×
Benzene	Air	2.95 × 10^−6^	1.04 × 10^−6^	4.32 × 10^−8^	−2.27 × 10^−7^
Ethylbenzene	Air	2.14 × 10^−6^		1.89 × 10^−8^	×
Butane	Air	1.64 × 10^−5^	1.15 × 10^−6^	4.18 × 10^−7^	×
Butene	Air	1.64 × 10^−6^	×	×	×
Ethane	Air	8.54 × 10^−6^	4.61 × 10^−7^	7.04 × 10^−8^	×
Ethanol	Air	1.54 × 10^−6^	×	×	×
Eten	Air	3.74 × 10^−5^	2.93 × 10^−7^	8.50 × 10^−8^	×
Formaldehyde	Air	5.67 × 10^−6^	5.03 × 10^−7^	2.53 × 10^−7^	×
Heptan	Air	4.48 × 10^−6^	2.76 × 10^−7^	1.36 × 10^−7^	×
Hexane	Air	8.71 × 10^−6^	7.42 × 10^−7^	2.79 × 10^−7^	×
Aliphatic hydrocarbons, alkanes, unspecified	Air	7.73 × 10^−6^	1.12 × 10^−6^	7.34 × 10^−8^	×
Aliphatic hydrocarbons, alkenes, unspecified	Air	4.77 × 10^−6^	2.64 × 10^−6^	×	×
Aromatic hydrocarbons	Air	2.92 × 10^−6^	1.68 × 10^−7^	1.43 × 10^−7^	2.08 × 10^−6^
Hydrocarbons, unspecified	Air	2.20 × 10^−7^	×	×	2.73 × 10^−6^
Methane	Air	7.90 × 10^−5^	4.52 × 10^−7^	4.33 × 10^−4^	−3.91 × 10^−5^
Methane, fossil	Air	1.12 × 10^−5^	1.71 × 10^−5^	2.12 × 10^−5^	×
NM VOC, non-methane volatile organic compounds	Air	2.59 × 10^−3^	1.81 × 10^−4^	5.01 × 10^−5^	−6.42 × 10^−4^
PAH	Air	4.92 × 10^−6^	×	1.82 × 10^−8^	−3.11 × 10^−7^
Pentane	Air	2.33 × 10^−5^	2.07 × 10^−6^	5.98 × 10^−7^	×
Propane	Air	1.03 × 10^−5^	7.69 × 10^−7^	2.18 × 10^−7^	×
Propene	Air	5.84 × 10^−6^	3.79 × 10^−7^	6.80 × 10^−8^	×
Toluene	Air	5.90 × 10^−6^	1.02 × 10^−6^	1.78 × 10^−7^	×
Xylene	Air	1.45 × 10^−5^	1.05 × 10^−5^	2.03 × 10^−7^	×
Unit	DALY	0.0029	0.0002	0.0005	−0.0007

**Table 5:** Results of studies characterizing the environmental consequences for inorganic compounds causing respiratory diseases at particular stages of the life cycle of the Vestas V90, DALY [own research].

Substance	Influence Area	Production	Use	Landfill	Recycling
Ammonia	Air	×	1.92 × 10^−4^	×	×
Nitric oxide	Air	3.90 × 10^−1^	8.53 × 10^−2^	1.73 × 10^−2^	−6.47 × 10^−2^
Solid particles	Air	1.82 × 10^−3^	2.44 × 10^−4^	×	−1.26 × 10^−2^
Solid particles, <10 µm (mobile)	Air	1.86 × 10^−2^	×	×	×
Solid particles, <10 µm (stationary)	Air	4.39 × 10^−1^	4.35 × 10^−4^	×	×
Solid particles, <2.5 µm	Air	2.59 × 10^−2^	9.99 × 10^−2^	1.23 × 10^−2^	×
Solid particles, >2.5 µm and <10 µm	Air	1.89 × 10^−2^	9.70 × 10^−3^	1.41 × 10^−3^	×
Sulfur dioxide	Air	3.00 × 10^−2^	1.36 × 10^−1^	4.22 × 10^−3^	×
Sulfur oxide	Air	6.40	6.71 × 10^−3^	×	−6.57 × 10^−2^
	DALY	7.330	0.340	0.036	−0.143

**Table 6:** The results of studies characterizing the environmental consequences for ionizing radiation occurring in the various stages of the life cycle of the Vestas V90, DALY [own research].

Substance	Influence Area	Production	Use	Landfill	Recycling
Carbon-14	Air	1.75 × 10^−3^	8.13 × 10^−5^	1.02 × 10^−4^	×
Iodine-129	Air	2.55 x 10^−5^	4.53 × 10^−7^	3.41 × 10^−7^	×
Krypton-85	Air	6.35 × 10^−5^	3.10 × 10^−7^	9.68 × 10^−12^	×
Polon-210	Air	×	2.63 × 10^−7^	×	×
Radon-222	Air	1.62 × 10^−2^	2.30 × 10^−4^	1.95 × 10^−4^	×
Uranium-238	Air	×	1.74 × 10^−7^	×	×
Cesium-134	Water	8.71 × 10^−5^	4.36 × 10^−7^	9.90 × 10^−9^	×
Cesium-137	Water	9.74 × 10^−4^	4.81 × 10^−6^	2.53 × 10^−7^	×
Cobalt-60	Water	1.19 × 10^−4^	6.08 × 10^−7^	1.49 × 10^−7^	×
Rad-226	Water	2.94 × 10^−5^	2.32 × 10^−7^	9.64 × 10^−8^	×
	DALY	0.0193	0.0003	0.0003	×

**Table 7:** The results of studies characterizing the environmental consequences for compounds that deplete the ozone layer, occurring at different stages of the life cycle of the Vestas V90, DALY [own research].

Substance	Influence Area	Production	Use	Landfill	Recycling
1,2-dichloro-1,1,2,2-tetrafluoroethane, CFC-114	Air	9.00 × 10^−5^	5.68 × 10^−7^	1.90 × 10^−7^	×
Bromochloro-difluoromethane, Halon 1211	Air	1.43 × 10^−6^	2.59 × 10^−6^	1.48 × 10^−7^	×
Bromotrifluoro	Air	1.05 × 10^−3^	1.18 × 10^−5^	5.31 × 10^−6^	−8.72 × 10^−5^
methane, Halon 1301	Air	×	1.74 × 10^−7^	×	×
Chlorodifluoro	Air	1.21 × 10^−5^	5.63 × 10^−7^	3.28 × 10^−10^	×
methane, HCFC-22	Air	2.89 × 10^−6^	1.17 × 10^−8^	2.95 × 10^−14^	×
Dichlorodifluoro	Air	1.16 × 10^−5^	1.97 × 10^−7^	2.63 × 10^−7^	×
methane, CFC-12	Air	4.00 × 10^−6^	1.94 × 10^−8^	1.20 × 10^−12^	×
	DALY	0.0012	0.0002	0.0001	−0.0001

**Table 8:** The results of the research characterizing the environmental consequences for the processes related to land use occurring in the various stages of the life cycle of the Vestas V90 wind farm, PDF m^2^/y [own research].

Substance	Influence Area	Production	Use	Landfill	Recycling
Use of class II-III land	Raw Materials	2.58 × 10^4^	1.30 × 10^2^	×	×
Use of class II-IV land	Raw Materials	3.25 × 10^3^	22.7	×	×
The use of class III-IV land	Raw Materials	3.19 × 10^3^	16.6	×	×
The use of class IV land	Raw Materials	3.75 × 10^2^		×	×
Occupation of agricultural land by non-irrigated areas	Raw Materials	×	44.1	×	×
Occupation by a construction area	Raw Materials		10	3.52 × 10^2^	×
Seizure by a landfill	Raw Materials	82.4	9.59 × 10^2^	2.12 × 10^3^	×
The occupation of an area of a commercial or normal forest	Raw Materials	3.78 × 10^2^	1.33 × 10^3^	15.4	×
Occupation by an industrial area	Raw Materials	55.1	4.74 × 10^2^	15.8	×
Occupation of built-up areas by an industrial area	Raw Materials	42.4	17.6	18.0	×
Occupation of an area with vegetation by an industrial area	Raw Materials	1.01 × 10^2^	15.2	31.9	×
Occupation by the area of extraction of mineral resources	Raw Materials	2.70 × 10^3^	2.50 × 10^2^	6.67 × 10^2^	×
Occupation of the area with sclerophyllous shrubs	Raw Materials	×	×	46.0	×
Occupation by communication areas, railway embankments	Raw Materials	×	31.4	×	×
Occupation by communication areas, rail network	Raw Materials	×	34.7	×	×
Occupation by communication areas, road embankments	Raw Materials	32.6	100	90.7	×
Occupation by traffic areas, road network	Raw Materials	78.5	96.1	2.65 × 10^3^	×
Convert non-irrigated land to arable land	Raw Materials	−1.83 × 10^2^	−2.44 × 10^2^	−68.0	×
Transformation of environmentally neutral waste landfills	Raw Materials	×	−30.6	×	×
Conversion of landfills for other waste	Raw Materials	×	−35.3	×	×
Conversion of sanitary waste landfills	Raw Materials	×	×	−2.10 × 10^3^	×
Conversion into a forest area	Raw Materials	−56.2	−80.6	−23.6	×
Transformation into an extensive forest area	Raw Materials	−9.02	−2.73 × 10^2^	−3.78	×
Transformation of the mining area of mineral resources	Raw Materials	−24.2	−2.39 × 10^2^	−5.88 × 10^2^	×
Transformation into an area of pastures and meadows	Raw Materials	−38.2	−86.0	−3.09 × 10^3^	×
Transformation into the area of seas and oceans	Raw Materials	−1.25 × 10^2^	−2.00 × 10^2^	−44.4	×
Transformation into an area with sclerophyllous shrubs	Raw Materials	×	−5.99	−2.76 × 10^2^	×
Transformation of other, undefined areas	Raw Materials	−1.12 × 10^4^	−1.15 × 10^3^	−2.87 × 10^3^	×
Conversion of arable land	Raw Materials	5.95 × 10^1^	3.12 × 10^2^	3.1	×
Conversion of irrigated arable land	Raw Materials	1.84 × 10^2^	2.44 × 10^2^	68.0	×
Transformation into landfills	Raw Materials	18,5	2.29 × 10^2^	4.22	×
Transformation into landfills affecting benthos	Raw Materials	1.25 × 10^2^	2.00 × 10^2^	44.3	×
Transformation into environmentally neutral waste landfills	Raw Materials	×	3.16	×	×
Conversion into a landfill for other waste	Raw Materials	×	35.3	×	×
Transformation into sanitary waste landfills	Raw Materials	×	×	2.10 × 10^3^	×
Transformation of the forest area	Raw Materials	3.13 × 10^2^	7.90	3.53 × 10^2^	×
Converting an area of a commercial or normal forest	Raw Materials	89.6	2.71 × 10^2^	3.61	×
Conversion into a heterogeneous agricultural area	Raw Materials	27.6	39.7	14.2	×
Conversion into an industrial area	Raw Materials	26.2	3.45 × 10^2^	2.91	×
Converting built-up areas into an industrial area	Raw Materials	26.4	12.0	15.1	×
Converting an area with vegetation into an industrial area	Raw Materials	61.2	11.1	21.9	×
Transformation into the area of extraction of mineral resources	Raw Materials	8.35 × 10^3^	8.98 × 10^2^	2.20 × 10^3^	×
Transformation of the area with sclerophyllous shrubs	Raw Materials	×	5.16	2.76 × 10^2^	×
Transformation into communication areas, railway embankments	Raw Materials	×	2.29	×	×
Transformation into communication areas, railway network	Raw Materials	×	2.5	×	×
Transformation into communication areas, road embankments	Raw Materials	×	20.3	×	×
Transformation into communication areas, road network	Raw Materials	36.0	39.4	4.31 × 10^2^	×
Transformation into other, undefined areas	Raw Materials	47.5	×	1.44	×
Transformation into artificial water reservoirs	Raw Materials	1.97 × 10^3^	68.2	5.09 × 10^2^	×
Transformation into artificial watercourses	Raw Materials	19.4	25.3	7.14	×

**Table 9:** The results of studies characterizing the environmental consequences for the processes related to the extraction of minerals occurring in the various stages of the life cycle of the Vestas V90, PDF m^2^/y [own research].

Substance	Influence Area	Production	Use	Landfill	Recycling
Aluminum, 24% Bauxite, 11% Raw Ore	Raw Materials	×	3.47 × 10^1^	×	×
Boksite, fossil	Raw Materials	8.08 × 10^4^	1.87 × 10^2^	×	−6.91 × 10^3^
Chrome, fossil	Raw Materials	4.95 × 10^4^	×	×	×
Copper, 0.99% sulfides, Cu 0.36% and Mo 8.2 x-3% crude ore	Raw Materials	×	2.71 × 10^1^	×	×
Copper, 1.18% sulfides, Cu 0.39% and Mo 8.2 x-3% crude ore	Raw Materials	×	1.49 × 10^2^	×	×
Copper, 1.42% sulfides, Cu 0.81% and Mo 8.2 x-3% crude ore	Raw Materials	×	3.96 × 10^1^	×	×
Copper, 2.19% sulfides, 1.83% Cu, and 8.2 x-3% Mo crude ore	Raw Materials	×	1.97 × 10^2^	×	×
Copper, fossil	Raw Materials	8.31 × 10^4^	2.76 × 10^4^	×	×
Iron ores, fossil	Raw Materials	9.18 × 10^−4^	×	×	−2.44 × 10^4^
Iron, 46% ore, 25% raw ore	Raw Materials	×	4.50 × 10^1^	×	×
Iron, fossil	Raw Materials	2.24 × 10^4^	1.79 × 10^2^	×	×
Molybdenum, 0.022% sulfides, Mo 8.2 x-3% and Cu 0.36% crude ore	Raw Materials	×	3.26 × 10^1^	×	×
Molybdenum, 0.11% sulfides, Mo 4.1 x-2% and Cu 0.36% crude ore	Raw Materials	×	6.57 × 10^1^	×	×
Nickel, 1.98% Silicates, 1.04% Crude Ore	Raw Materials	7.54 × 10^2^	5.95 × 10^2^	5.86 × 10^2^	×
Fossil nickel	Raw Materials	1.04 × 10^6^	×	×	×
	PDF·m^2^/r	1.279.121	29.134	1.043	−31.277

**Table 10:** The results of the study characterizing the environmental consequences for the processes related to the extraction of non-renewable energy, occurring at individual stages of the life cycle of the Vestas V90, MJ wind farm [own research].

Substance	Influence Area	Production	Use	Landfill	Recycling
Coal, 18 MJ/kg, fossil	Raw Materials	1.15 × 10^5^	6.48 × 10^2^	×	−5.35 × 10^4^
Fossil coal, unspecified, fossil	Raw Materials	5.04 × 10^3^	3.20 × 10^4^	2.07 × 10^2^	×
Gas production processes linked to coal extraction/kg	Raw Materials	3.75 × 10^4^	2.20 × 10^2^	×	×
Gas extraction processes associated with coal extraction/m^3^	Raw Materials	1.25 × 10^3^	1.02 × 10^4^	6.49 × 10^1^	×
Natural gas, 35 MJ/m^3^, fossil	Raw Materials	6.11 × 10^5^	2.72 × 10^3^	×	8.59 × 10^3^
Natural gas, 36.6 MJ/m^3^, fossil fuel	Raw Materials	3.07 × 10^3^	4.12 × 10^2^	×	−3.98 × 10^4^
Natural gas, raw material, 35 MJ/m^3^, fossil fuel	Raw Materials	×	×	×	−5.95 × 10^4^
Natural gas, fossil gas	Raw Materials	1.94 × 10^5^	4.37 × 10^4^	8.21 × 10^3^	
Crude oil, 42.6 MJ/kg, fossil	Raw Materials	1.30 × 10^6^	5.45 × 10^3^	×	−1.73 × 10^5^
Crude oil, raw material, 41 MJ/kg, fossil	Raw Materials	×	×	×	−5.61 × 10^4^
Petroleum, fossil	Raw Materials	2.00 × 10^5^	1.14 × 10^5^	5.80 × 10^4^	×
	PDF·m^2^/r	2.469.917	209.483	66.463	−373.783

## Data Availability

Not applicable.
